# Nrf2-Mediated Pathway Activated by *Prunus spinosa* L. (Rosaceae) Fruit Extract: Bioinformatics Analyses and Experimental Validation

**DOI:** 10.3390/nu15092132

**Published:** 2023-04-28

**Authors:** Mariastella Colomba, Serena Benedetti, Daniele Fraternale, Andrea Guidarelli, Sofia Coppari, Valerio Freschi, Rita Crinelli, George E. N. Kass, Andrea Gorassini, Giancarlo Verardo, Carla Roselli, Maria Assunta Meli, Barbara Di Giacomo, Maria Cristina Albertini

**Affiliations:** 1 Department of Biomolecular Sciences (DiSB), University of Urbino Carlo Bo, 61029 Urbino, Italy; mariastella.colomba@uniurb.it (M.C.); serena.benedetti@uniurb.it (S.B.); daniele.fraternale@uniurb.it (D.F.); andrea.guidarelli@uniurb.it (A.G.); s.coppari3@campus.uniurb.it (S.C.); rita.crinelli@uniurb.it (R.C.); carla.roselli@uniurb.it (C.R.); maria.meli@uniurb.it (M.A.M.); barbara.digiacomo@uniurb.it (B.D.G.); 2 Department of Pure and Applied Sciences (DiSPeA), University of Urbino Carlo Bo, 61029 Urbino, Italy; valerio.freschi@uniurb.it; 3 European Food Safety Authority (EFSA), 43126 Parma, Italy; georges.kass@efsa.europa.eu; 4 Department of Humanities and Cultural Heritage, University of Udine, 33100 Udine, Italy; andrea.gorassini@uniud.it; 5 Department of Agriculture, Food, Environment and Animal Sciences, University of Udine, 33100 Udine, Italy; giancarlo.verardo@uniud.it

**Keywords:** antioxidant activity, blackthorn, Chemprop, miRNet, oxidative stress, phenolic compounds

## Abstract

In our previous studies, *Prunus spinosa* fruit (PSF) ethanol extract was showed to exert antioxidant, antimicrobial, anti-inflammatory and wound healing activities. In the present study, an integrated bioinformatics analysis combined with experimental validation was carried out to investigate the biological mechanism(s) that are responsible for the reported PSF beneficial effects as an antioxidant during a pro-inflammatory TLR4 insult. Bioinformatics analysis using miRNet 2.0 was carried out to address which biological process(es) the extract could be involved in. In addition, Chemprop was employed to identify the key targets of nuclear receptor (NR) signaling and stress response (SR) pathways potentially modulated. The miRNet analysis suggested that the PSF extract mostly activates the biological process of cellular senescence. The Chemprop analysis predicted three possible targets for nine phytochemicals found in the extract: (i) ARE signaling, (ii) mitochondrial membrane potential (MMP) and (iii) p53 SR pathways. The PSF extract antioxidant effect was also experimentally validated in vitro using the human monocyte U937 cell line. Our findings showed that Nrf2 is modulated by the extract with a consequent reduction of the oxidative stress level. This was confirmed by a strong decrease in the amount of reactive oxygen species (ROS) observed in the PSF-treated cells subjected to lipopolysaccharide (LPS) (6 h treatment, 1 µg/mL). No visible effects were observed on p53 and MMP modulation.

## 1. Introduction

Free radicals are by-products of many cellular metabolic processes. However, cells produce antioxidants to neutralize them, and in general, normal physiological levels of oxidative stress are controlled by the body’s self-defense mechanisms [1]. Once this equilibrium, known as redox state, is disrupted, oxidative stress occurs causing mild inflammation, which can lead to cell and tissue damage. Uncontrolled oxidative stress can accelerate the aging process and may contribute to the development of several conditions, including chronic diseases [2,3]. Phenolic antioxidants, vitamins and other natural phytochemicals are known to reduce oxidative stress leading to the protection of cells against its adverse effects [4,5].

*Prunus spinosa* L. (Rosaceae family) (common name, blackthorn) is a wild plum shrub/small tree which is commonly found on the slopes of wide uncultivated areas in Europe and in deciduous forests and temperate countries in Asia [6]. *P. spinosa* fruits contain a combination of bioactive phytochemicals, which make them a potential source of nutraceuticals. The beneficial effects of *P. spinosa* are probably due to the polyphenols present in the plant, which include flavonoids, anthocyanins, phenolic acids, flavonols, tannins and ascorbic acid [7,8]. Considering this interesting chemical composition with beneficial potentials on humans [9], we already tested the extract for its antioxidant, anti-inflammatory and anti-bacterial properties [10]. We also evaluated the anti-aging capacity and the role of the extract in wound closure [11]. Recently, we compared the effect of the *P. spinosa* fruit (PSF) extract, both free and encapsulated within 1,2-dioleoyl-sn-glycero-3-phosphocholine (DOPC) and 1,2-dioleoyl-sn-glycero-3-phospho-(10-rac-glycerol) (DOPG) nanoparticles, on the endothelium LPS-mediated inflammation. Our findings showed a preferential incorporation of the extract within DOPC vesicles with a total amount of phenolic compounds, which was three times higher than that observed in PSF-DOPG vesicles [12].

As over the past few decades, a great amount of information related to molecular biology has been produced, and biological data must be combined to form a comprehensive picture, we decided to try a bioinformatic approach to further investigate the action mechanism(s) by which the PSF extract exerts its beneficial effects. We have therefore used Chemprop (downloaded from https://github.com/Chemprop on 1 December 2022), a message passing neural network for molecular property prediction, and miRNet 2.0 (freely available at https://www.mirnet.ca7, accessed on 1 June 2021), a web-based platform designed to help to elucidate microRNA (miRNA) functions or small compounds/target genes interactions by integrating users’ data with existing knowledge via network-based visual analytics.

All PSF extract phenolic compounds detected by HPLC-DAD-ESI-MS^n^ were analyzed by Chemprop given as input in the form of smiles strings. Unfortunately, the analysis by the miRNet was partial as, among all PSF phenolics, only two (“anthocyanin” and “quercetin”) were present within the miRNet “small compounds” list. Chemprop analyses predicted that nine out of twenty phenolic compounds found in the extract could be involved in three different targets including (i) Keap-1/Nrf2/ARE signaling pathway, (ii) mitochondrial membrane potential modulation and (iii) p53 modulation. The miRNet retrieved the “cellular senescence” process as the most likely activated by the extract, in line with a previous report [11].

It is widely known that polyphenols can directly neutralize reactive oxygen species (ROS) and act through a mechanism mediated by Nrf2 (Nuclear factor E2-related factor 2) that leads the activation of the transcription of cytoprotective genes (phase II detoxifying genes) and the production of antioxidant enzymes to confer protection against oxidative stress. Moreover, replicative senescence of cells has been reported to be delayed by Nrf2 [13].

Nrf2 represents a crucial regulator of the cellular defense mechanisms against xenobiotics and oxidative stress. At the same time, it is involved in cancer prevention and progression [14,15,16]. Under unstressed conditions, Nrf2 is bound to the repressor protein Keap1, which anchors Nrf2 in the cytoplasm and targets it for ubiquitination and proteasome degradation. Upon exposure to stressors, the Nrf2/Keap1 complex dissociates and Nrf2 translocates to the nucleus. The mechanisms underlying the activation of the Keap1/Nrf2/ARE pathway are not well understood but may involve oxidation/alkylation of key thiol(s) in Keap1 and/or phosphorylation of Nrf2. Upon exposure to electrophiles or oxidants, the cysteine-rich protein Keap1 is modified by forming covalent adduct(s) or disulfide bond(s) within the protein, and it is this cysteine-based modification of Keap1 that allows the dissociation of Nrf2 from Keap1 and subsequent nuclear translocation of Nrf2 [17,18].

Protein p53 is a transcription factor made up of 393 amino acids and a molecular weight of 53 kDa. It might be a key coordinator of oxidative stress and aging. In response to low levels of oxidative stimuli, p53 exhibits antioxidant activities to eliminate oxidative stress and ensure cell survival; in response to high levels of oxidative insults, p53 exhibits pro-oxidative activities that further increase the levels of stress, leading to cell death. Protein p53 accomplishes these context-dependent roles by regulating the expression of a panel of genes involved in cellular responses to oxidative insults and by modulating other pathways important for oxidative stress responses. The mechanism that switches p53 function from antioxidant to pro-oxidant remains unclear. Nevertheless, several polyphenols have shown therapeutic benefits against oxidative stress diseases by suppressing p53-dependent apoptosis [19].

Mitochondria are considered the major source of ROS in the cell and the accumulation of ROS-associated damages may cause progressive cell dysfunctions. A loss of mitochondrial membrane potential (MMP) is a signal of bioenergetic stress and may result in the release of apoptotic factors leading to cell death. Some dietary polyphenols have been reported to have a protective effect by preventing the decrease of MMP and improving mitochondrial function [20,21]. On this basis, plant polyphenols are generally considered as antioxidants, and thus candidates for the role of mitochondria-protecting agents [22,23].

In the previous studies [10,11,12], we proved that the PSF extract anti-inflammatory effect involves the modulation of the TLR4 signaling pathway. In the present study, we explored more in detail the action mechanism(s) by which the extract exerts its antioxidant effect during a pro-inflammatory TLR4 stimulation. To this aim, an in vitro experimental protocol using human monocytes (U937 cells) was set up to confirm the results obtained by the bioinformatics analysis.

## 2. Materials and Methods

### 2.1. Extract Preparation

The plants were identified, by morphological analysis, by Professor Daniele Fraternale. The *P. spinosa* fruit (PSF) ethanol extract used in this study was obtained as described in [11,12]. Briefly, 50 g of ripe fruit pulp and peel of *P. spinosa* collected in November 2019 at the locality “la Caputa” Urbania (PU, Marche region, Italy), GPS coordinates 43°40′48.5″ N, 12°31′53.3″ E (350 m above sea level), and stored frozen at −20 °C until use, were homogenized in Osterizer for 3 min in 50 mL of acidified 70% aqueous ethanol solution (70 mL ethanol/29.9 mL distilled H_2_O/0.1 mL HCl 36%). After filtration of the homogenate with a Buchner filter under vacuum, the residue of the first extraction was re-extracted in the same way, and the two supernatants obtained were combined and centrifuged at 10,000× *g* for 15 min. The supernatant obtained after centrifugation was then dried under vacuum in a Büchi rotavapor at 37 °C. The total amount of dried PSF extract from 50 g of fruit pulp and peel of *P. spinosa* ripe fruits was 11.6 g. The dried sample was stored at −20 °C until use.

### 2.2. Determination of the Phenolic Composition of P. spinosa Extract by HPLC-DAD-ESI-MS^n^

#### 2.2.1. Chemicals and Materials

Methanol (MeOH), formic acid (HCOOH), gallic acid, chlorogenic acid, cyanidin chloride and phloridzin were purchased from Sigma-Aldrich (Milan, Italy). Quercetin-3-*O*-galactoside was obtained from ExtraSynthese (Lyon, France). Quercetin-3-*O*-arabinoside, quercetin-3-*O*-xyloside and quercetin-3-*O*-rhamnoside were purchased from Carbosynth (Berkshire, UK). Milli-Q^®^ grade water was produced by the Elgastat UHQ-PS system (ELGA, HighWycombe Bucks, UK). Solid phase extraction (SPE) columns ISOLUTE C18, 1 g, 6 mL were from Biotage (Milan, Italy).

#### 2.2.2. SPE Purification

Each dried sample (10 mg) was diluted with 2 mL of deionized water and loaded on a C18 SPE column previously conditioned with 10 mL MeOH containing 0.4% HCOOH and 10 mL H_2_O containing 0.4% HCOOH.

After loading, the column was washed with 12 mL of 0.4% formic acid in the water, and the phenolic fraction was eluted with 5 mL of MeOH with 0.4% of formic acid. The solvents were removed under vacuum (T < 35 °C), and the residue was diluted with 1 mL H_2_O/MeOH 9:1 (*v*/*v*) and analyzed by HPLC-DAD-ESI-MS^n^.

#### 2.2.3. HPLC-DAD-ESI-MS^n^ Analysis

The procedure for MS^n^ identification and quantification of bioactive compounds found in *P. spinosa* fruit ethanol extract used in this study has been previously described in our work [12]. Briefly, a UHPLC Ultimate 3000 (Thermo Scientific, San Jose, CA, USA) was coupled in parallel with a Finnigan LXQ linear ion trap mass spectrometer (Thermo Scientific, San Jose, CA, USA) and a diode array detector (DAD; Thermo Scientific, San Jose, CA, USA) by splitting the mobile phase 1:1. The ion source was an electrospray ionization source (ESI) operating both in negative and positive modes at the following conditions: transfer line capillary at 275 °C; ion spray voltage at 3.30 kV; sheath, auxiliary and sweep gas (N_2_) flow rates at 50, 10 and 0 arbitrary units, respectively. Helium was used as the collision-damping gas in the ion trap set at a pressure of 0.13 Pa. The acquisition was carried out in full scan (*m*/*z* 50–1500) and in full scan MS^2^ (*m*/*z* 50–800). ESI-MS^2^ spectra were obtained by collision-induced dissociation experiments after the isolation of the appropriate precursor ion in the ion trap. The chromatographic separation was performed with a column Synergi Hydro, 4 μm, 250 × 2.0 mm (Phenomenex, Castel Maggiore, Italy), thermostated at 30 °C. Elution was carried out at a flow rate of 0.3 mL/min, using as a mobile phase a mixture of 0.2% formic acid in methanol (A) and 0.2% formic acid in water (B) with the following gradient: 0–6 min 10% A, 20 min 40% A, 40 min 40% A, 46 min 100% A, 56 min 100% A, 58 min 10% A and 58–65 min 10% A. The injection volume was 20 μL. The phenolic compounds identification was performed by comparing the fragmentation pattern of each component with standards and/or with data available in the literature. 

The quantitative analysis was carried out using an Ultimate 3000 RS Diode Array Detector controlled by Chromeleon software (version 6.80). The quantification of the phenolic compounds was carried out by external calibration from the areas of the chromatographic peaks obtained by UV detection at the following wavelengths: 520 nm for anthocyanins, 328 nm for hydroxycinnamic acid derivatives, 280 nm for hydroxybenzoic acid derivatives and 258 nm for flavones and flavonols. A stock solution of each standard in H_2_O/MeOH (9:1, *v*/*v*) with 0.4% formic acid was diluted with the same solvent to prepare calibration curves ranging from 12 to 3000 ng/mL. The R^2^ coefficients for the calibration curves were >0.99. When standards were unavailable, the quantification of the analytes was carried out using the calibration curve of available standards presenting similar chemical structures. The samples were analyzed in triplicate.

### 2.3. The miRNet Analysis

The miRNet (http://www.mirnet.ca, accessed on 1 June 2021) is an easy-to-use web platform designed for understanding the functions of microRNAs (miRNAs) and interactions between miRNAs or small compounds and target genes [24]. In the past decade, significant progress has been made in discovering the functionality of miRNAs and identifying their targets, and it has been shown that miRNAs can influence most biological processes. Considering the large amount of data and that each miRNA can modulate different genes, miRNet is based on the integration of data from 11 databases including miRTarBase, TarBase, miRecords, SM2miR, Pharmaco-miR, miR2Disease, PhenomiR, StarBase, EpimiR, miRDB and miRanda. The program features an interactive flowchart to allow users to choose an analysis path based on their input. The input can be loaded in three different ways: by entering a list of IDs (miRNA, genes, lncRNA), by loading a data table (qRT-PCR, microarray or RNA-seq) from miRNA/mRNA expression studies or by selecting from a list of available database entries (disease names, small molecules and epigenetic modifiers). The key property of miRNet is the network visualization system associated with the enrichment analysis. These features derive from the integration of different combinations capable of providing important biological information; in fact, the program supports functional annotations based on database of GO, KEGG or Reactome paths. Hypergeometric tests can be performed to reveal the functional profiles of genes that are modulated by miRNAs. Through this integrative approach, users can easily identify key miRNA targets to gain a better understanding of the collective role of multiple miRNAs and their gene targets. From the main page of the miRNet site, it is possible to choose the “small compounds” item and select one or more compounds from the list. Among the main compounds of the extract, only the items “anthocyanin” and “quercetin” were explored as the only ones available within the miRNet “small compounds” list (https://www.mirnet.ca/miRNet/upload/MoleculeUploadView.xhtml accessed on 1 June 2021). All miRNAs obtained from miRNet analyses were used to visualize associated target genes. Since the displayed network size was too large (edge 8653), we used grade filters and selected only genes with a grade greater than or equal to 5 to perform our analysis. We applied conventional enrichment analysis, then hypergeometric testing on the highlighted genes using Reactome.

### 2.4. Chemprop Analysis

To predict some basic properties of the PSF extract, we adopted a machine-learning approach based on neural networks. More specifically, we used Chemprop, a directed-message-passing neural network (D-MPNN) that has recently demonstrated state-of-the-art performance in the task of predicting different chemical properties of small molecules across a wide range of data [25]. D-MPNN is a particular type of neural network that takes as input a molecule description (provided, for instance, according to the so-called smiles strings format) [26] and encodes this information as a graph with atoms representing nodes and bonds representing edges. This type of neural network works in two phases: (i) a message-passing phase, during which information is sent across the molecule with the purpose of building its neural representation; (ii) a readout phase that exploits the achieved representation to infer specific properties of interest about the molecule. Feature vectors associated with atoms are updated during the message-passing phase, based on their neighbors’ features and the related bonds. The resulting representation of the molecule learned by the model during the first phase can finally be used in the readout phase to perform predictions by means of a feedforward neural network that enables the learning of the relation between molecule encoding and specific output properties.

With the aim of gaining some predictive knowledge regarding the properties of *P. spinosa* fruit extract, we trained Chemprop on the Tox21 dataset. Tox21 is a publicly accessible database targeting the measurements of the toxicity of compounds and/or the identification of mechanisms of action for further investigation, that has been created within the “Toxicology in the 21st Century” (Tox21) initiative (https://tripod.nih.gov/tox21/challenge/ accessed on 1 December 2022) and has been used as a benchmark for several data challenges and scientific works [25,27]. It consists of qualitative toxicity measurements for about 10,000 unique molecules evaluated according to 12 different targets of nuclear receptor (NR) signaling and stress response (SR)pathways. The targets explored by Tox21 are summarized in Table 1.

Given the availability, for every molecule of the dataset, of the compound activity outcome (active or inactive) in one or more of the 12 pathway assays, we used Tox21 data to train a D-MPNN model with the following default configuration values: ReLU nonlinear activation functions, batch size = 50, depth = 3; number of training epochs = 30, ffn_hidden_size: 300, ffn_num_layers: 2.

The resulting model was then used to perform inference regarding each of the 12 classification tasks for the twenty molecules found in the extract given as input in the form of canonical or isomeric smiles strings.

### 2.5. Cell Culture

U937 human myeloid leukemia cells (Sigma-Aldrich, Milan, Italy) were cultured in suspension in RPMI 1640 medium (Sigma-Aldrich, Milan, Italy). Culture media were supplemented with 10% fetal bovine serum (Euroclone, Celbio Biotecnologie, Milan, Italy), penicillin (100 units/mL) and streptomycin (100 mg/mL) (Euroclone, Celbio Biotecnologie, Milan, Italy). Cells were grown at 37 °C in T-75 tissue culture flasks (Corning Inc., Corning, NY, USA) gassed with an atmosphere of 95% air—5% CO_2_.

### 2.6. Cell Viability

Cells (5 × 10^5^) were grown in 35 mm tissue culture, exposed in complete RPMI 1640 culture medium for 18 h to vehicle (EtOH 0.005%) or 40 µg/mL PSF extract and analyzed with the trypan blue exclusion assay. Briefly, an aliquot of the cell suspension was diluted 1:2 (*v*/*v*) with 0.4% trypan blue, and the necrotic cells (i.e., those including trypan blue) were counted using the hemocytometer. To analyze the apoptotic cells, after the treatment, cells were incubated for 5 min with 10 μM Hoechst 33342 (Sigma-Aldrich). The cells were finally analyzed with a fluorescence microscope to assess their nuclear morphology for chromatin condensation and fragmentation. Cells with homogeneously stained nuclei were considered viable. The percentage of viable cells was evaluated as follows:

% necrotic/apoptotic cells = number of necrotic or apoptotic cells/total cell number × 100.

### 2.7. Fluorescence Analysis of Cells Treated with P. spinosa Extract

U937 cells (5 × 10^5^) were counted and pelleted by centrifugation (500 rpm for 2 min). The pellets were washed with PBS and treated with 40 µg/mL of PSF extract for 10 min. Following the treatment, an aliquot of the cell suspension was placed on a coverslip and the fluorescence emission by the cells was evaluated using a BX-51 microscope (Olympus, Milan, Italy), equipped with a SPOT-RT camera unit (Diagnostic Instruments, Delta Sistemi, Rome, Italy) using an Olympus LCAch 40x/0.55 objective lens. The excitation and emission wavelengths were 380 and 500 nm with a 5 nm slit width for both emission and excitation.

### 2.8. Measurement of Mitochondrial Membrane Potential (MMP)

Cells (5 × 10^5^) were cultured in 35 mm tissue culture plates containing an uncoated coverslip, exposed for 30 min with 50 nM MitoTracker Red CMXRos (Thermo Fisher Scientific, Milan, Italy) before the end of treatment with the PSF extract (18 h). After the treatment, the cells were centrifuged and incubated for 10 min in 2 mL of saline A (8.182 g/L NaCl, 0.372 g/L KCl, 0.336 g/L NaHCO_3_ and 0.9 g/L glucose, pH 7.4), which makes the cells to attach to the coverslip. 

The cells were finally washed three times in phosphate buffer saline (PBS, 136 mM NaCl, 10 mM Na_2_HPO_4_, 1.5 mM KH_2_PO_4_, 3 mM KCl; pH 7.4), and the fluorescence images were visualized using a fluorescence microscope. The excitation and emission wavelengths were 545 and 610 nm, respectively, with a 5 nm slit width for both emission and excitation. Mean fluorescence values were determined by averaging the fluorescence values of at least 50 cells/treatment condition/experiment. 

### 2.9. Chloromethyl-2′,7′-dichlorodihydrofluorescein Diacetate (DCF) Fluorescence Assay

To evaluate PSF antioxidant activity, cells were stimulated with 1 µg/mL Lipopolysaccharide (LPS, Sigma-Aldrich) for 1, 6 and 18 h (with or without 40 µg/mL of plum extract). Briefly, cells (5 × 10^5^) were exposed in 35 mm tissue culture dishes for increasing time intervals to vehicle (EtOH 0.005%) and PSF in the absence or presence of LPS. After the treatments, the cells were washed with saline A and incubated for 30 min with 20 µM DCF (Thermo Fisher Scientific, Milan, Italy) in culture medium without fetal bovine serum. Subsequently, the cells were washed three times with PBS, and the fluorescence images were visualized using a fluorescence microscope. The excitation and emission wavelengths were 488 and 515 nm, with a 5 nm slit width for both emission and excitation. Images were collected with exposure times of 100–400 ms, digitally acquired and processed for fluorescence determination at the single cell level by ImageJ software. Mean fluorescence values were determined by averaging the fluorescence values of at least 50 cells/treatment condition/experiment.

### 2.10. Cell Extract Preparation and Western Immunoblotting Analysis

After treatment, cells (2 × 10^6^) were washed with ice-cold PBS and then lysed in a denaturing buffer consisting of 50 mM Tris-HCl pH 7.8, 0.25 M sucrose, 2% (*w*/*v*) sodium dodecyl sulphate (SDS) and 10 mM N-ethylmaleimide, supplemented before use with a cocktail of protease inhibitors (Complete Tablets, Roche, Mannheim, Germany) as previously described [28]. Lysates were heated at 100 °C and then sonicated at 70 Watt for 40 s to reduce viscosity. Cell debris was removed by centrifugation at 14,000× *g*, and protein content was determined in the cleared extracts by the Lowry assay, using bovine albumin as standard.

Whole cell extracts were loaded onto polyacrylamide gels and proteins were resolved by SDS-PAGE (PolyAcrylamide Gel Electrophoresis, Bio-Rad, Segrate, Italy) and then transferred to a PolyVinylDenFluoride (PVDF) membrane. To demonstrate equal protein loading, total proteins were stained on the blots with the No-stain Protein Labeling Reagent (A44449), obtained from Invitrogen, according to the manufacturer’s instructions. The fluorescent signal was acquired in a Chemidoc MP Imaging System (Bio-Rad, Hercules, CA, USA). Membranes were blocked in 5% (*w*/*v*) non-fat dry milk and incubated overnight at 4 °C with the following primary antibodies: rabbit polyclonal anti-Nrf2 (D1Z9C, XP#12721, 1:1000) and mouse monoclonal anti-p53 (1C12, #2524, 1:1000) from Cell Signaling Technology. Detection was performed with a goat anti-rabbit or anti-mouse IgG HRP-conjugated (Bio-Rad, Segrate, Italy) and the enhanced chemiluminescence kit WesternBright (Advansta, San Jose, CA, USA). The signal was detected in an imaging system as above.

### 2.11. Nrf2 Activity Assay

U937 cells (2 × 10^6^) were incubated for 1, 6 and 18 h with the vehicle (0.005% EtOH) or 40 µg/mL PSF extract in the absence or presence of 1 µg/mL LPS. At each experimental time point, cells were washed with PBS, and nuclear and cytoplasmic proteins were extracted by the RayBio^®^ Nuclear Extraction Kit (RayBiotech, Norcross, GA, USA), following the manufacturer’s instructions. Thereafter, Nrf2 activity was evaluated in the protein extracts by the RayBio^®^ NRF2 TF-Activity Assay (RayBiotech, Norcross, GA, USA) and a non-radioactive transcription factor assay with an ELISA format in which double-stranded oligonucleotides containing an Nrf2 binding sequence (5′-TGACTCAG-3′) were coated in 96-well plates. After incubations with the primary antibody against the Nrf2-DNA complex and the HRP-conjugated secondary antibody, the signal was obtained through a spectrophotometric plate reader at 450 nm (Thermo Fisher Scientific, Milan, Italy).

### 2.12. Statistical Analysis

GraphPad Prism was used for statistical analysis. All quantitative data were presented as the mean ± standard deviation (SD). Statistically significant differences were obtained using one-way ANOVA. A *p* value of < 0.05 was considered statistically significant.

## 3. Results

### 3.1. Phenolic Content of P. spinosa Extract

The HPLC-DAD-ESI-MS^n^ analysis of P. spinosa fruit ethanol extract used in this study highlighted a composition quite similar to the one described in our previous work [12]. P. spinosa extract phenolic compounds, their content (μg/g dw) and MS^n^ characterization are reported in Table 2 and Table S1 (Supplementary Materials), respectively.

### 3.2. The miRNet Analysis of P. spinosa Extract

Only two items (anthocyanin and quercetin) out of the twenty identified PSF phenolic compounds were available in the “small compounds” miRNet list (see https://www.mirnet.ca/miRNet/upload/MoleculeUploadView.xhtml accessed on 1 June 2021). After selecting the “anthocyanin” item, the network revealed the involvement of 19 miRNAs: hsa-mir-1-3p, hsa-mir-190a-5p, hsa-mir-335-5p, hsa-mir-429, hsa-mir-450a-5p, hsa-mir-486-5p, hsa-mir-532-5p, hsa-let-7b-3p, h3.3sa-mir-30b-3p, hsa-mir-30c-1-3p, hsa-mir-298, hsa-mir-1, hsa-mir-190a, hsa-mir-190, hsa-mir-335, hsa-mir-450a, hsa-let-7b, hsa-mir-30b and hsa-mir-30c-1.

While selecting the entry “quercetin”, the network recovered two miRNAs: hsa-mir-99b-5p and hsa-mir-99b. The interaction network between the twenty-one miRNAs putatively modulated by anthocyanin and quercetin was used to visualize the associated target genes (thirty-five) which are listed in Table S2 (Supplementary Materials).

We applied conventional enrichment analysis, then hypergeometric test on the highlighted genes using Reactome. The most significant Reactome pathway recovered was “cellular senescence” (*p* value = 0.0263), confirming what we already reported in a previous study [11].

### 3.3. Chemprop Analysis of Phenolic Compounds of P. spinosa Extract

The phenolic molecules analyzed along with the results of the inference task performed with the Chemprop neural network trained on Tox21 are reported in Table 3.

For each of the polyphenolic molecules, the corresponding score obtained by the neural network model is reported. Higher prediction values can be associated with positive outcomes of the classification task. By setting a threshold (*th*) = 0.5, all entries above *th* can be classified as positive and all values below *th* as negative ones. As shown in Table 3, we found some molecules predicted by the neural network to be (putatively) classified as belonging to the corresponding class (i.e., biological pathway).

### 3.4. Internalization of P. spinosa Extract Quercetin Derivatives

**Figure 1:**
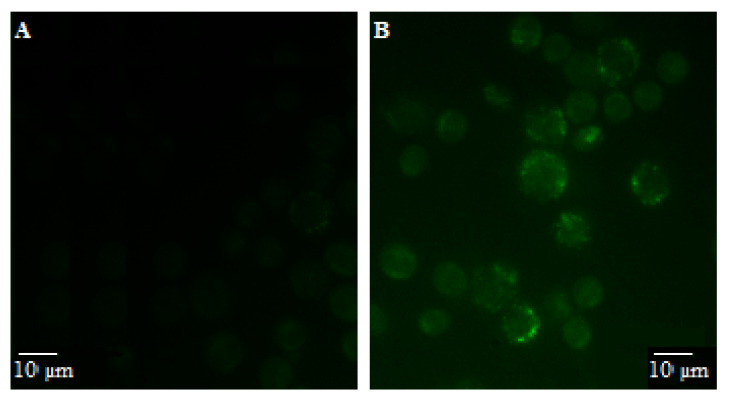
Internalization of quercetin derivatives of the extract. Representative microscope images of U937 cells treated for 10 min with the vehicle (**A**) or 40 µg/mL PSF extract (**B**).

Quercetin and its derivatives can emit intrinsic fluorescence in living cells, with a maximum excitation of about 370 nm and emission of about 500 nm; this is due to non-covalent bonds with some intracellular targets (probably proteins) [29]. Since the PSF extract is characterized by several quercetin derivatives involved in Chemprop predictions [12] (Table 2 and Table 3), we verified the internalization of these compounds by the fluorescence emission in U937 cells used for all the other experimental investigations. As indicated in Figure 1, it is possible to notice point fluorescence, corresponding to the mitochondrial compartment, that diffuses in the short term (after 10 min, Figure 1B) fading a few (nearly 10) minutes later.

### 3.5. Mitochondrial Membrane Potential (MMP) Evaluation

Following bioinformatics analysis prediction about a possible MMP modification, the variation of the mitochondrial membrane potential in the treated cells was also evaluated. No evident variations were observed. In addition, when considering cell viability, apoptosis (CTR = 2 ± 1%; LPS = 1 ± 1%; PSF = 2 ± 1%; PSF + LPS = 1 ± 1% apoptotic cells) and necrosis (CTR = 3 ± 1%; LPS = 2 ± 1%; PSF = 3 ± 1%; PSF + LPS = 1 ± 1% necrotic cells) were never detected different in treated samples compared to controls.

### 3.6. Evaluation of the Antioxidant Activity (DCF Analysis)

*P. spinosa* extract antioxidant activity was evaluated by measuring the production of ROS, mainly hydrogen peroxide (H_2_O_2_) by DCF analysis. This assay is characterized by the proportion between the shift in fluorescence intensity and the amount of ROS produced within the cells. Four experimental groups were set up: C (controls, i.e., U937 cells receiving the vehicle (0.005% EtOH)), L (cells treated with LPS, 1 µg/mL), P (cells treated with plum extract, 40 µg/mL) and P + L (cells treated with plum extract and LPS, 40 µg/mL and 1 µg/mL, respectively).

**Figure 2:**
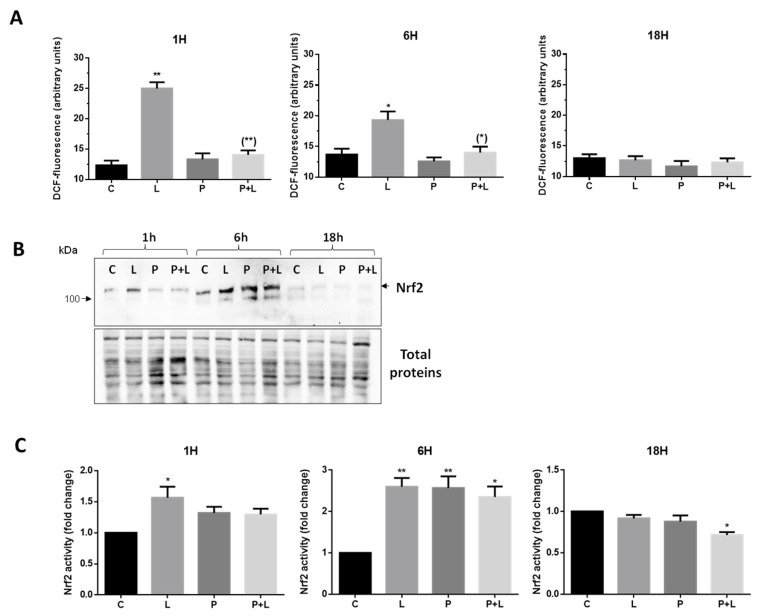
(**A**) U937 cells were treated for 1, 6, or 18 h with the vehicle (C), 1 µg/mL of LPS (L) and 40 µg/mL PSF extract in the absence (P) or presence of 1 µg/mL LPS (P + L). After treatments, the cells were analyzed for DCF-fluorescence. The results are represented as means ± SD calculated from at least three distinct experiments. (**B**) Western immunoblotting analysis of Nrf2 levels in whole cell extracts. U937 cells were treated for 1, 6, or 18 h with vehicle (C), 1 µg/mL of LPS (L), plum extract 40 µg/mL in the absence (P) or presence of 1 µg/mL LPS (P + L). Proteins (20 µg) were resolved by 8% (*w*/*v*) SDS-PAGE, immunoblotted and Nrf2 levels were detected with a specific antibody (upper panel). Total proteins were stained as loading control (lower panel). (**C**) Analysis of Nrf2 activity in U937 nuclear protein extracts (10 µg) from the same experimental groups reported above. Nuclear proteins were extracted by the RayBio^®^ Nuclear Extraction Kit and Nrf2 activity was evaluated in the protein extracts by the RayBio^®^ NRF2 TF-Activity Assay. Data are represented as means ± SD calculated from at least three independent experiments. * *p* < 0.05 and ** *p* < 0.01 vs. untreated cells (ANOVA followed by Dunnett’s test). (*) *p* < 0.05 and (**) *p* < 0.01 as compared to cells treated with LPS (ANOVA followed by Dunnett’s test).

As shown in Figure 2A, LPS administration induced a significant increment of ROS production both at 1 and 6 h of treatment (maximum at 1 h), while in the presence of PSF, extract ROS levels were comparable to those of the untreated control even in LPS-exposed cells. At 18 h, no significant differences were observed in the three experimental conditions (L, P, P + L) when compared to controls.

### 3.7. Nrf2 Modulation by P. spinosa Extract

To validate the results obtained in silico, the expression level of Nrf2 and p53 was evaluated by Western immunoblotting analysis of whole cell lysates obtained from cells incubated under the treatment conditions described above. Indeed, since both factors are activated by protein stabilization, their relative levels reflect their activation state. In line with the viability tests, in which cells treated with *P. spinosa* do not become neither necrotic nor apoptotic, p53 levels were undetectable, suggesting that, at least in all the experimental conditions tested the extract did not produce any toxicity. By contrast, the increased Nrf2 levels compared to control cells were detected after 1 h of LPS stimulation and further increased at 6 h (Figure 2B). At this time point, Nrf2 accumulation was also observed in cells treated with PSF extract alone and in combination with LPS. In all cases, Nrf2 activation was transient as demonstrated by signal intensity returning to control levels at 18 h incubation (Figure 2B).

Based on the qualitative results of the immunoblotting analysis, Nrf2 activation was next quantified in U937 nuclear extracts by a non-radioactive transcription factor assay with an ELISA format. As reported in Figure 2C, a significant increment of Nrf2 activity was evidenced in LPS-stimulated cells (L) both after 1 and 6 h treatments, when compared to untreated control cells (C). The plum extract alone (P) or the extract plus LPS (P + L) displayed significantly increased Nrf2 activity only at 6 h of incubation. After 18 h, Nrf2 activity was not incremented in LPS- and PSF-exposed cells. 

## 4. Discussion

As a continuation of previous studies already published [10,11,12], in the present paper, we focused on exploring more in detail the mechanism(s) by which the PSF extract exerts its antioxidant effect related to its functional activities. HPLC-DAD-ESI-MS^n^ showed that the PSF phenolic composition included twenty compounds belonging to the following classes: (i) hydroxycinnamic acid derivatives, (ii) anthocyanins, (iii) hydroxybenzoic acid derivatives and (iv) flavonoid derivatives (Table 2). To investigate the molecular mechanisms (putatively) responsible for PSF functional activities, bioinformatics analyses were carried out, and the predicted results were experimentally validated in vitro.

Analysis by miRNet addressed “cellular senescence” as the process most probably activated by the extract, in line with what we already reported in a previous study. Particularly, in that study HUVECs were maintained in long-term cultures (160 days) until growth arrest to mimic cellular aging, with cultures being supplemented with PSF ethanol extract at different concentrations (20, 40 and 80 μg/mL) for the entire experimental period. Although the extract did not delay the onset of aging phenotype, nevertheless it appeared to have a qualitative effect on cell differentiation, at least at higher concentrations. In fact, senescent cells (P15) treated with PSF at 40 and 80 μg/mL showed a younger morphology and, when considering inflammation markers, miR-146a, IL-6 and IRAK-1 expression levels were comparable to those observed in younger cells (P3). These findings were interpreted hypothesizing that the biological effect of the extract could cope with the proinflammatory phenotype associated with cellular senescence. In the same study, the PSF antioxidant effect was confirmed also by in vivo treatments in *C. elegans*; moreover, a beneficial effect of the extract was observed on worms’ lifespan and health span with positive outcomes on longevity markers (i.e., miR-124 upregulation and miR-39 downregulation) [11].

Chemprop analysis (Table 3) suggested that nine out of twenty polyphenols found in the extract might be involved in three biological targets, (i) ARE signaling pathway, (ii) mitochondrial membrane potential stress and (iii) p53 stress response.

As most quercetin derivatives appeared involved in all the three biological targets, their cellular internalization was examined. Obtained results highlighted that these compounds can enter the cell to carry out their biological activity. Furthermore, in line with other studies [30], PSF quercetin derivatives could accumulate in the intramitochondrial compartment. As localization in the mitochondria is functional to the scavenging of ROS, largely generated in this compartment [31], the antioxidant property was also measured. Our findings confirmed a significant (maximum after 1 h), although transient (no visible effects at 18 h), antioxidant activity of the extract.

Additional in vitro experimental tests using human monocytes U937 cells suggested that the extract seems to exert its antioxidant effect (revealed by a significant decrease of ROS levels) by modulating Nrf2 both level and activity. No effects were observed on p53 and MMP variation at the concentration (40 µg/mL) employed which, apparently, does not cause any mitochondrial dysfunction (cells were always vital and neither apoptosis nor necrosis was ever observed).

When considering the modulation of Nrf2, our findings are in line with numerous other studies where the capacity of phenolic compounds to interact with biological systems modulating, for example, gene expression has been one of the mechanisms postulated to explain part of the health benefits [32,33]. From a chemical point of view, phenolic compounds are molecules with a single or more phenyl rings bearing one or more hydroxyl groups. The presence of phenolic groups capable of reducing reactive oxygen species confers redox properties and, in vitro, most of these compounds display strong antioxidant activities [34,35] inducing endogenous antioxidant defense mechanisms by modulating transcription factors such as the Nrf2, which is a central regulator of cellular resistance to oxidative damage and a therapeutic target in aging-related diseases [36,37]. For this reason, phenolic compounds have traditionally been considered useful in the prevention of chronic diseases associated with oxidative stress (e.g., cardiovascular and neurodegenerative diseases, diabetes and various types of cancer) [18,38,39].

Western blotting output confirmed the activation of Nrf2, most likely due to PSF polyphenols, in cells treated for 6 h with the extract, thus corroborating what observed in other cell lines [18]. Based on the HPLC-DAD-ESI-MS^n^ analysis, the major phenolic compounds present in the PSF extract are phenolic acids, followed by anthocyanins and flavonoids. All these compounds are reported as Nrf2 activators [40,41,42,43,44,45]. More specifically, 3-*O*-caffeoylquinic acid (3-CQA) has a concentration that represents nearly half of the total phenol content (4003.53 ± 16.17 out of 8269.33 ± 41.54 µg/g dw). Caffeoylquinic acids (CQAs), esters of caffeic acid with quinic acid, are specialized bioactive metabolites derived from the phenylpropanoid biosynthesis pathway. In humans, CQAs have been reported to show a wide range of potential benefits with therapeutic applications due to their antioxidant, antimicrobial, anticancer and neuroprotective activity [38]. CQAs can play roles as direct antioxidants (scavenging free radicals) or act as Michael acceptors and target the Keap1/Nrf2 pathway [46]. Quercetin has been reported to upregulate Nrf2 protein expression in different human cell lines, although at concentrations higher than physiologically relevant concentrations [47,48,49,50]. Moreover, orally administered quercetin was stated to upregulate Nrf2 protein levels against LPS-stimulated intestinal oxidative stress in broiler chickens [51].

Many subclasses of flavonoids promote Nrf2 nuclear translocation [52,53]. These data are absolutely in line with our observation that Nrf2 activity increased in nuclear extracts of cells treated for 6 h with the PSF extract, confirming its translocation from the cytosol to the nucleus. In addition, interesting findings on the upregulation of Nrf2 nuclear translocation by polyphenols have been reported in several pre-clinical studies [51,54,55,56,57].

In the present study, we observed the activation of Nrf2 either in physiological conditions (P, 6 h (Figure 2C) or in LPS-treated cells, where the extract re-establishes the physiological levels of ROS (P + L, 6 h; Figure 2A)). The observation that Nrf2 activity increases on its own by the extract may be explained by the polyphenols’ nature. In fact, (dietary) polyphenolic antioxidants can often relieve Nrf2 repression by Keap1. Decreased levels of ROS observed in PSF and LPS treated cells (P + L) are in line with previous results reporting that quercetin activates Nrf2/ARE and restores redox homeostasis in both cellular and animal models subjected to oxidative stress inducers [51]. Moreover, the extract is alleged to indirectly interfere with the activation of the LPS receptor TLR4 by a negative feedback loop mechanism activated by miR-146a targeting IRAK-1 (which is down regulated) and leading to a decreased expression of inflammation markers [11].

Although further data are certainly necessary to better define the action mechanism(s) by which the PSF extract exerts its beneficial health effects, the PSF-mediated inhibition of the TLR4/NF-kB biological pathway and the PSF-mediated Nrf2 modulation are in line with available data reporting that various phenolic compounds with protective effects against oxidative stress and inflammation can act either on NF-kB or Nrf2 transcription factors [39] and references therein.

While the evidence for the antioxidant activity of the PSF extract under in vitro conditions is compelling, additional work would be needed to explore this activity in other in vivo experimental models where the PSF extract is administered orally. Crucial here would be human data demonstrating the antioxidant activity of the extract and, most importantly, data supporting the postulated beneficial properties of PSF following a full risk–benefit analysis.

## 5. Conclusions

The PSF extract plays a cytoprotective (antioxidant) role by decreasing ROS levels during inflammation when Nrf2 is activated and can subsequently prevent a pro-inflammatory response by inhibiting the TLR4/NF-kB-mediated inflammatory cascade, which can explain all the functional activities observed in our previous studies on PSF extract bioeffects, including the wound healing and anti-aging properties previously reported.

## Figures and Tables

**Table 1:** Chemprop targets including nuclear receptor (NR) signaling and stress response (SR) pathways and their related description.

No.	Targets	Description
1	NR-AR	assay to identify small molecule agonists of the androgen receptor (AR) signaling pathway using the MDA cell line
2	NR-AR-LBD	assay to identify small molecule agonists of the androgen receptor (AR) signaling pathway
3	NR-AhR	assay to identify small molecules that activate the aryl hydrocarbon receptor (AhR) signaling pathway
4	NR-Aromatase	assay to identify aromatase inhibitors
5	NR-ER	assay to identify small molecule agonists of the estrogen receptor alpha (ER-alpha) signaling pathway using the BG1 cell line
6	NR-ER-LBD	assay to identify small molecule agonists of the estrogen receptor alpha (ER-alpha) signaling pathway
7	NR-PPAR-gamma	assay to identify small molecule agonists of the peroxisome proliferator-activated receptor gamma (PPARg) signaling pathway
8	SR-ARE	assay for small molecule agonists of the antioxidant response element (ARE) signaling pathway
9	SR-ATAD5	assay for small molecules that induce genotoxicity in human embryonic kidney cells expressing luciferase-tagged ATAD5
10	SR-HSE	assay for small molecule activators of the heat shock response signaling pathway
11	SR-MMP	assay for small molecule disruptors of the mitochondrial membrane potential
12	SR-p53	assay for small molecule agonists of the p53 signaling pathway

**Table 2:** Phenolic content (μg/g dw and percentage) of *P. spinosa* extract determined by HPLC-DAD-ESI-MS^n^.

No	Compound	Content (μg/g dw)	Mean Content (%)
1	3-*O*-Caffeoylquinic acid (3-CQA)	4003.53 ± 16.17	48.41
2	3-*O*-p-Coumaroylquinic acid (3-p-CoQA)	199.83 ± 2.19	2.42
3	Caffeoylquinic acid dehydrodimer	39.21 ± 0.24	0.47
4	3-*O*-Feruloylquinic acid (3-FQA)	196.73 ± 2.43	2.38
5	4-*O*-Caffeoylquinic acid (4-CQA)	299.96 ± 1.98	3.63
6	Caffeoylquinic acid dehydrodimer isomer	134.21 ± 2.03	1.62
	∑ Hydroxycinnamic acid derivatives	4873.42 ± 26.12	58.93
7	Cyanidin 3-*O*-glucoside	564.77 ± 6.80	6.83
8	Cyanidin 3-*O*-rutinoside	856.68 ± 9.16	10.36
9	Peonidin 3-*O*-glucoside	203.47 ± 1.18	2.46
10	Peonidin 3-*O*-rutinoside	752.20 ± 10.47	9.10
	∑ Anthocyanins	2377.12 ± 6.67	28.75
11	4-(vanilloyloxy)-2,6,6-trimethylcyclohexene-1-carboxylic acid	7.83 ± 0.15	0.09
	∑ Hydroxybenzoic acid derivatives	7.83 ± 0.15	0.09
12	Apigenin pentoside	6.05 ± 0.08	0.07
13	Apigenin pentoside isomer	10.00 ± 0.19	0.12
14	Quercetin-hexoside-pentoside	75.05 ± 1.15	0.91
15	Rutin	167.49 ± 1.07	2.03
16	Quercetin 3-*O*-galactoside	222.91 ± 3.44	2.70
17	Quercetin 3-*O*-xyloside	64.54 ± 0.78	0.78
18	Quercetin 3-*O*-arabinoside	84.47 ± 0.56	1.02
19	Quercetin pentoside	284.72 ± 1.12	3.44
20	Quercetin 3-*O*-rhamnoside	95.75 ± 0.32	1.16
	∑ Flavonoid derivatives	1010.97 ± 8.60	12.23
	Total phenolic compounds	8269.33 ± 41.54	

Data are expressed as the mean value ± standard deviation; *n* = 3 repetitions. The average percentage content for each phenolic compound was calculated as follows: mean content (%) = content of the single phenolic compound (µg/gr dw)/content of the total phenolic compounds (µg/gr dw) × 100.

**Table 3:** Prediction scores for *P. spinosa* ethanol extract small molecules on the Tox21 classification targets. All the targets of nuclear receptor (NR) signaling and stress response (SR) pathways are indicated. All entries with threshold (*th*) > 0.5 are classified as positive (in bold).

No	Compound	NR-AR	NR-AR-LBD	NR-AhR	NR-Aromatase	NR-ER	NR-ER-LBD	NR-PPAR-gamma	SR-ARE	SR-ATAD5	SR-HSE	SR-MMP	SR-p53
1	**3-*O*-Caffeoylquinic acid (3-CQA)**	0.0532	0.0376	0.0502	0.0132	0.1047	0.0563	0.1382	0.1536	0.0801	0.0630	0.0620	0.1135
2	**3-*O*-p-Coumaroylquinic acid (3-p-CoQA)**	0.1437	0.1448	0.0215	0.0427	0.2578	0.0927	0.1310	0.2224	0.0963	0.0519	0.1232	0.1615
3	**Caffeoylquinic acid dehydrodimer**	0.0567	0.0508	0.0681	0.0344	0.1281	0.0703	0.2012	0.1889	0.1135	0.0801	0.1115	0.2238
4	**3-*O*-Feruloylquinic acid (3-FQA)**	0.1070	0.0874	0.0333	0.0284	0.1441	0.0530	0.1064	0.1739	0.0833	0.0497	0.0779	0.1357
5	**4-*O*-Caffeoylquinic acid (4-CQA)**	0.0654	0.0636	0.0546	0.0290	0.1437	0.0851	0.1920	0.2689	0.1382	0.0993	0.1301	0.2166
6	**Caffeoylquinic acid dehydrodimer isomer**	0.0567	0.0508	0.0681	0.0344	0.1281	0.0703	0.2012	0.1889	0.1135	0.0801	0.1115	0.2238
7	**Cyanidin 3-*O*-glucoside**	0.0705	0.0826	0.2448	0.1242	0.3776	0.2138	0.0850	0.4916	0.1196	0.0986	**0.6072**	**0.5227**
8	**Cyanidin 3-*O*-rutinoside**	0.0319	0.0348	0.1342	0.0545	0.2239	0.1055	0.0837	0.3123	0.0979	0.0571	0.1884	0.3870
9	**Peonidin 3-*O*-glucoside**	0.0707	0.0771	0.2316	0.1255	0.3334	0.1616	0.0890	0.4232	0.1151	0.0799	**0.5015**	0.4953
10	**Peonidin 3-*O*-rutinoside**	0.0460	0.0533	0.0892	0.0745	0.2964	0.1192	0.0764	0.2642	0.0918	0.0448	0.2428	0.4113
11	**4-(vanilloyloxy)-2,6,6-trimethylcyclohexene-1-carboxylic acid**	0.0149	0.0061	0.0826	0.0364	0.0798	0.0504	0.0578	0.1316	0.0368	0.0775	0.2620	0.0619
12	**Apigenin pentoside**	0.0595	0.0764	0.2636	0.1116	0.3539	0.1957	0.2096	0.4552	0.2228	0.1288	0.4121	**0.5178**
13	**Apigenin pentoside isomer**	0.0595	0.0764	0.2636	0.1116	0.3539	0.1957	0.2096	0.4552	0.2228	0.1288	0.4121	**0.5178**
14	**Quercetin hexoside-pentoside**	0.0325	0.0316	0.1042	0.0398	0.1810	0.0859	0.0843	0.2824	0.0914	0.0495	0.1301	0.3472
15	**Rutin**	0.0790	0.0885	0.0538	0.0705	0.4809	0.2301	0.0569	0.2308	0.0768	0.0366	0.3834	0.4153
16	**Quercetin 3-*O*-galactoside**	0.0771	0.1047	0.2179	0.1346	0.4228	0.2718	0.0977	0.4850	0.1404	0.1121	**0.6302**	**0.5602**
17	**Quercetin 3-*O*-xyloside**	0.0785	0.1257	0.3414	0.1941	0.4162	0.2803	0.1449	**0.6347**	0.2174	0.1848	**0.7389**	**0.6537**
18	**Quercetin 3-*O*-arabinoside**	0.0705	0.1062	0.2916	0.1449	0.4227	0.3137	0.1225	**0.5764**	0.1832	0.1581	**0.6973**	**0.6104**
19	**Quercetin pentoside**	0.0494	0.0731	0.4738	0.1447	0.2836	0.1910	0.1928	**0.6568**	0.2507	0.2220	**0.6121**	**0.6019**
20	**Quercetin 3-*O*-rhamnoside**	0.0744	0.0999	0.2808	0.1601	0.4283	0.2713	0.1035	**0.5510**	0.1595	0.1420	**0.7367**	**0.5956**

## Data Availability

Data is contained within the article or Supplementary Materials.

## Supplementary Materials

The following supporting information can be downloaded at: https://www.mdpi.com/article/10.3390/nu15092132/s1, Table S1: ESI–MS^n^ characterization of phenolic compounds of *Prunus spinosa* ethanolic extract purified samples; Table S2: Genes modulated by the twenty-one (21) miRNAs obtained according to miRNet analysis of the items “anthocyanins” and “quercetin” as the only two (out of all PSF polyphenols) available within the “small compounds” list.
