# Supplementary material for: Nrf2-Mediated Pathway Activated by Prunus spinosa L. (Rosaceae) Fruit Extract: Bioinformatics Analyses and Experimental Validation
Source: Nutrients. 2023 Apr 28;15(9):2132. doi: 10.3390/nu15092132 (PMC10181019; doi:10.3390/nu15092132)
Supplement: Supplementary file 1 [file nutrients-15-02132-s001.zip › nutrients-2356121-supplementary (1).pdf]

**Table S1:** ESI–MS$^n$ characterization of phenolic compounds of *Prunus spinosa* ethanolic extract purified samples.

| No | $M^+$ (*m/z*) | $[M-H]^-$ or $[M+HCOO]^-$ (*m/z*) | HPLC-ESI/MS$^n$ *m/z* (% base peak) | Tentative assignment | Ref. |
|---|---|---|---|---|---|
| 1 | | 353 | MS$^2$[353]: 191 (100), 179 (44), 135 (8) | 3-*O*-Caffeoylquinic acid | [12] |
| 2 | | 337 | MS$^2$[337]: 163 (100), 191 (9), 173 (6) | 3-*O*-p-Coumaroylquinic acid | [12] |
| 3 | | 705 | MS$^2$[705]: 513 (100); MS$^3$ [705 $\rightarrow$ 513]: 339 (100); MS$^4$ [705 $\rightarrow$ 513 $\rightarrow$ 339]: 295 (100) | Caffeoylquinic acid dehydrodimer | [12] |
| 4 | | 367 | MS$^2$[367]: 193 (100), 134 (5), 173 (3), 191 (2) | 3-*O*-Feruloylquinic acid | [12] |
| 5 | | 353 | MS$^2$[353]: 173 (100), 179 (54), 191 (28), 135 (65) | 4-*O*-Caffeoylquinic acid | [12] |
| 6 | | 705 | MS$^2$[705]: 513 (100); MS$^3$ [705 $\rightarrow$ 513]: 339 (100); MS$^4$ [705 $\rightarrow$ 513 $\rightarrow$ 339]: 295 (100) | Caffeoylquinic acid dehydrodimer isomer | [12] |
| 7 | 449 | | MS$^2$[449]: 287 (100) | Cyanidin 3-*O*-glucoside | [12] |
| 8 | 595 | | MS$^2$[595]: 287 (100), 449 (20) | Cyanidin 3-*O*-rutinoside | [12] |
| 9 | 463 | | MS$^2$[463]: 301 (100) | Peonidin 3-*O*-glucoside | [12] |
| 10 | 609 | | MS$^2$[609]: 301 (100), 463 (30) | Peonidin 3-*O*-rutinoside | [12] |
| 11 | | 333 | MS$^2$[333]: 165 (100), 301 (30), 289 (10) | 4-(vanilloyloxy)-2,6,6-trimethylcyclohexene-1-carboxylic acid | [12] |
| 12 | | 447[a] | MS$^2$[447]: 401 (100); MS$^3$ [447 $\rightarrow$ 401]: 269 (100) | Apigenin pentoside | [12] |
| 13 | | 447[a] | MS$^2$[447]: 401 (100); MS$^3$ [447 $\rightarrow$ 401]: 269 (100) | Apigenin pentoside isomer | [12] |
| 14 | | 595 | MS$^2$[595]: 300 (100), 415 (40), 301 (40), 271 (18), 505 (30), 433 (12) | Quercetin-hexoside-pentoside | [12] |
| 15 | | 609 | MS$^2$[609]: 301 (100), 343 (10) | Rutin | [12] |
| 16 | | 463 | MS$^2$[463]: 301 (100) | Quercetin 3-*O*-galactoside | b |
| 17 | | 433 | MS$^2$[433]: 301 (100) | Quercetin 3-*O*-xyloside | b |
| 18 | | 433 | MS$^2$[433]: 301 (100), 300 (80) | Quercetin 3-*O*-arabinoside | b |
| 19 | | 433 | MS$^2$[433]: 301 (100) | Quercetin pentoside | [12] |
| 20 | | 447 | MS$^2$[447]: 301 (100), 300 (20), 285 (10) | Quercetin 3-*O*-rhamnoside | b |

[a]Formate adduct. [b]Confirmed with standard.

**Table S2:** Genes modulated by the twenty-one (21) miRNAs obtained according to miRNet analysis of the items “anthocyanins” and “quercetin” as the only two (out of all PSF polyphenols) available within the
“small compounds” list.

| No | Gene | Description |
|---|---|---|
| 1 | **ZNF264** | zinc finger protein 264 |
| 2 | **ZNF460** | zinc finger protein 460 |
| 3 | **ABI2** | abl interactor 2 |
| 4 | **PLAGL2** | PLAG1 like zinc finger 2 |
| 5 | **SP1** | Sp1 transcription factor |
| 6 | **PDCD10** | programmed cell death 10 |
| 7 | **BRI3BP** | BRI3 binding protein |
| 8 | **HMGA1** | high mobility group AT-hook 1 |
| 9 | **LDLR** | low density lipoprotein receptor |
| 10 | **MARCHF6** | E3 ubiquitin ligase membrane-associated ring-CH-type finger 6 |
| 11 | **HNRNPU** | heterogeneous nuclear ribonucleoprotein U |
| 12 | **IGF1** | insulin like growth factor 1 |
| 13 | **ITGA3** | integrin subunit alpha 3 |
| 14 | **SRSF7** | serine and arginine rich splicing factor 7 |
| 15 | **SSR1** | signal sequence receptor subunit 1 |
| 16 | **YWHAZ** | tyrosine 3-monooxygenase/tryptophan 5-monooxygenase activation protein zeta |
| 17 | **NFAT5** | nuclear factor of activated T cells 5 |
| 18 | **FOXP1** | forkhead box P1 |
| 19 | **FBXO45** | F-box protein 45 |
| 20 | **CDKN1B** | cyclin dependent kinase inhibitor 1B |
| 21 | **TNRC6A** | trinucleotide repeat containing adaptor 6A |

| | | |
|---|---|---|
| 22 | **ARID3A** | AT-rich interaction domain 3A |
| 23 | **IGF1R** | insulin like growth factor 1 receptor |
| 24 | **CRKL** | CRK like proto-oncogene, adaptor protein |
| 25 | **MSMO1** | methylsterol monooxygenase 1 |
| 26 | **CACNG8** | calcium voltage-gated channel auxiliary subunit gamma 8 |
| 27 | **XKR4** | XK related 4 |
| 28 | **LIN28B** | lin-28 homolog B |
| 29 | **LRRC3C** | leucine rich repeat containing 3C |
| 30 | **RIF1** | replication timing regulatory factor 1 |
| 31 | **UBXN2B** | UBX domain protein 2B |
| 32 | **CCNF** | cyclin F |
| 33 | **KHSRP** | KH-type splicing regulatory protein |
| 34 | **KLHDC10** | kelch domain containing 10 |
| 35 | **SLC38A7** | solute carrier family 38 member 7 |
